# Need for Contraceptive Services Among Women of Reproductive Age — 45 Jurisdictions, United States, 2017–2019

**DOI:** 10.15585/mmwr.mm7025a2

**Published:** 2021-06-25

**Authors:** Lauren B. Zapata, Karen Pazol, Kathryn M. Curtis, Debra J. Kane, Tara C. Jatlaoui, Suzanne G. Folger, Ekwutosi M. Okoroh, Shanna Cox, Maura K. Whiteman

**Affiliations:** ^1^Division of Reproductive Health, National Center for Chronic Disease Prevention and Health Promotion, CDC; ^2^Division of Human Development and Disability, National Center on Birth Defects and Developmental Disabilities, CDC; ^3^Iowa Department of Public Health; ^4^Immunization Services Division, National Center for Immunization and Respiratory Diseases, CDC.

Ensuring access to contraceptive services is an important strategy for preventing unintended pregnancies, which account for nearly one half of all U.S. pregnancies (*1*) and are associated with adverse maternal and infant health outcomes (*2*). Equitable, person-centered contraceptive access is also important to ensure reproductive autonomy (*3*). Behavioral Risk Factor Surveillance System (BRFSS) data collected during 2017–2019 were used to estimate the proportion of women aged 18–49 years who were at risk for unintended pregnancy* and had ongoing or potential need for contraceptive services.^†^ During 2017–2019, in the 45 jurisdictions^§^ from which data were collected, 76.2% of women aged 18–49 years were considered to be at risk for unintended pregnancy, ranging from 67.0% (Alaska) to 84.6% (Georgia); 60.7% of women had ongoing or potential need for contraceptive services, ranging from 45.3% (Puerto Rico) to 73.7% (New York). For all jurisdictions combined, the proportion of women who were at risk for unintended pregnancy and had ongoing or potential need for contraceptive services varied significantly by age group, race/ethnicity, and urban-rural status. Among women with ongoing or potential need for contraceptive services, 15.2% used a long-acting reversible method (intrauterine device or contraceptive implant), 25.0% used a short-acting reversible method (injectable, pill, transdermal patch, or vaginal ring), and 29.5% used a barrier or other reversible method (diaphragm, condom, withdrawal, cervical cap, sponge, spermicide, fertility-awareness–based method, or emergency contraception). In addition, 30.3% of women with ongoing or potential need were not using any method of contraception. Data in this report can be used to help guide jurisdictional planning to deliver contraceptive services, reduce unintended pregnancies, ensure that the contraceptive needs of women and their partners are met, and evaluate efforts to increase access to contraception.

BRFSS is a state-based, random-digit-dialed telephone survey that collects self-reported health information from U.S. adults.^¶^ This report uses the most recent available data for the optional Family Planning Module** (2017 data for seven jurisdictions and 2019 data for 38 jurisdictions). The proportion of women aged 18–49 years at risk for unintended pregnancy was estimated.^††^ Women were considered to be at risk for unintended pregnancy unless they reported 1) not being sexually active with a male partner, 2) being currently pregnant or seeking pregnancy, 3) not minding being pregnant, or 4) having had a hysterectomy. This approach is consistent with prior evaluation of BRFSS contraceptive use data (*4*). The proportion and total number of women aged 18–49 years with ongoing or potential need for contraceptive services, defined as women considered to be at risk for unintended pregnancy who were not using permanent contraception (female sterilization or male partner vasectomy), were also estimated.^§§^ Estimates were calculated overall, by jurisdiction, and by selected sociodemographic characteristics.^¶¶^ Chi-square tests were conducted to examine differences in distributions by sociodemographic characteristics.

Among women with ongoing or potential need for contraceptive services, the proportions who were using a specific contraceptive method at last sexual encounter were estimated*** by category of method or no method, overall, and by jurisdiction. Categories of contraceptive methods reflect different levels of effort for method initiation and continuation. Long-acting reversible contraception methods include intrauterine devices and contraceptive implants; these methods require the most clinical effort for initiation but require minimal follow-up until time for removal or reinsertion and minimal action by the woman. Short-acting reversible contraception methods include injectables, pills, transdermal patches, and vaginal rings; these methods require less clinical effort for initiation than long-acting reversible methods but require ongoing clinical services and supplies and action by the woman to maintain use. Barrier or other reversible methods included diaphragms, condoms (male or female), withdrawal, cervical caps, sponges, spermicides, fertility-awareness–based methods, and emergency contraception; these methods have little or no need for clinical services for initiation but require action by the woman or her partner to maintain use. Analyses were conducted using SAS (version 9.4; SAS Institute) with SUDAAN to account for the complex sampling methods used in BRFSS; data were weighted for nonresponse.^†††^

Among women aged 18–49 years in the 45 jurisdictions, 76.2% were considered to be at risk for unintended pregnancy, ranging from 67.0% (Alaska) to 84.6% (Georgia); 60.7% had ongoing or potential need for contraceptive services, ranging from 45.3% (Puerto Rico) to 73.7% (New York) (Table 1). For all jurisdictions combined, the proportion of women who were at risk for unintended pregnancy and had need for contraceptive services varied significantly by age group, race/ethnicity, and urban-rural status (Table 2). Although these proportions did not vary by insurance coverage or routine checkup within the past year, among women with ongoing or potential need for contraceptive services, 16.7% did not have insurance, and 27.4% did not have a routine checkup within the past year.

**TABLE 1:** Estimated numbers and percentages of women aged 18–49 years who were at risk for unintended pregnancy* and had ongoing or potential need for contraceptive services,^†^ by jurisdiction — Behavioral Risk Factor Surveillance System, 45 jurisdictions, 2017–2019^§^

Jurisdiction	Women aged 18–49 yrs			
	Total no.^¶^	% at risk for unintended pregnancy (95% CI)	No. and % who had ongoing or potential need for contraceptive services	
			No. (95% CI)^¶^	% (95% CI)
Alabama	1,005,000	76.8 (73.5–79.8)	571,800 (533,700–610,000)	56.9 (53.1–60.7)
Alaska	146,300	67.0 (60.6–72.9)	75,600 (65,400–85,700)	51.7 (44.7–58.6)
Arizona	1,444,100	80.9 (76.6–84.5)	875,100 (804,400–943,000)	60.6 (55.7–65.3)
Arkansas	596,100	75.3 (70.5–79.5)	347,500 (316,500–377,900)	58.3 (53.1–63.4)
California	8,514,600	70.6 (67.6–73.5)	5,091,700 (4,810,700–5,355,700)	59.8 (56.5–62.9)
Connecticut	694,700	82.7 (79.6–85.3)	481,400 (455,700–505,000)	69.3 (65.6–72.7)
Delaware	189,900	79.6 (74.0–84.3)	125,300 (114,100–135,800)	66.0 (60.1–71.5)
District of Columbia	192,700	73.9 (69.5–78.0)	131,600 (122,700–139,900)	68.3 (63.7–72.6)
Florida	4,130,200	80.4 (76.4–83.9)	2,589,600 (2,403,800–2,767,200)	62.7 (58.2–67.0)
Georgia	2,226,700	84.6 (81.0–87.6)	1,572,100 (1,474,100–1,661,100)	70.6 (66.2–74.6)
Hawaii	275,300	70.8 (67.5–73.8)	164,400 (154,400–174,000)	59.7 (56.1–63.2)
Idaho	350,100	77.9 (73.7–81.7)	188,000 (170,100–205,500)	53.7 (48.6–58.7)
Illinois	2,604,400	78.6 (75.1–81.7)	1,669,400 (1,567,800–1,763,200)	64.1 (60.2–67.7)
Indiana	1,349,100	75.7 (72.6–78.6)	785,200 (739,300–831,000)	58.2 (54.8–61.6)
Iowa	613,400	79.4 (77.0–81.6)	348,400 (330,600–365,600)	56.8 (53.9–59.6)
Kansas	573,400	77.6 (74.9–80.0)	342,900 (325,700–358,900)	59.8 (56.8–62.6)
Louisiana	968,400	79.8 (76.3–82.9)	625,600 (586,900–662,400)	64.6 (60.6–68.4)
Maine	244,500	71.5 (66.8–75.8)	131,300 (118,800–143,800)	53.7 (48.6–58.8)
Maryland	1,214,500	77.0 (74.2–79.5)	763,900 (727,500–800,400)	62.9 (59.9–65.9)
Massachusetts	1,420,600	80.1 (76.9–83.0)	964,600 (913,400–1,012,900)	67.9 (64.3–71.3)
Minnesota	1,119,500	78.9 (76.9–80.8)	690,700 (665,000–715,400)	61.7 (59.4–63.9)
Mississippi	610,100	75.4 (71.7–78.8)	338,600 (313,600–362,400)	55.5 (51.4–59.4)
Missouri	1,222,900	78.9 (75.4–82.0)	694,600 (644,500–743,500)	56.8 (52.7–60.8)
Montana	203,400	80.6 (77.4–83.4)	115,900 (108,200–123,500)	57.0 (53.2–60.7)
Nebraska	380,800	80.1 (76.9–83.0)	243,300 (229,200–256,700)	63.9 (60.2–67.4)
Nevada	619,000	73.7 (68.2–78.6)	359,000 (322,500–394,300)	58.0 (52.1–63.7)
New Jersey	1,792,400	74.9 (71.4–78.1)	1,100,500 (1,032,400–1,166,900)	61.4 (57.6–65.1)
New Mexico	409,000	81.0 (77.2–84.3)	246,600 (228,200–264,200)	60.3 (55.8–64.6)
New York	4,069,700	81.6 (77.1–85.4)	2,999,400 (2,800,000–3,178,400)	73.7 (68.8–78.1)
North Carolina	2,155,000	71.3 (67.1–75.2)	1,204,600 (1,109,800–1,299,500)	55.9 (51.5–60.3)
Ohio	2,281,900	78.1 (74.0–81.8)	1,360,000 (1,255,000–1,460,400)	59.6 (55.0–64.0)
Oklahoma	788,500	75.6 (70.5–80.0)	414,000 (371,400–455,000)	52.5 (47.1–57.7)
Oregon	861,400	82.0 (78.8–84.8)	528,900 (495,300–560,800)	61.4 (57.5–65.1)
Pennsylvania	2,446,600	77.7 (73.9–81.1)	1,460,600 (1,357,900–1,560,900)	59.7 (55.5–63.8)
Rhode Island	215,300	80.3 (75.4–84.5)	144,700 (133,500–155,200)	67.2 (62.0–72.1)
South Carolina	1,010,300	79.3 (75.8–82.4)	640,500 (599,100–678,900)	63.4 (59.3–67.2)
South Dakota	170,200	81.4 (76.4–85.5)	103,100 (92,900–112,700)	60.6 (54.6–66.2)
Tennessee	1,362,800	78.4 (74.2–82.0)	800,000 (735,900–861,300)	58.7 (54.0–63.2)
Texas	6,093,500	67.4 (63.1–71.5)	3,199,100 (2,918,800–3,479,400)	52.5 (47.9–57.1)
Utah	707,100	75.4 (73.2–77.6)	403,000 (386,100–420,700)	57.0 (54.6–59.5)
Virginia	1,759,700	78.9 (75.7–81.7)	1,113,900 (1,050,500–1,173,700)	63.3 (59.7–66.7)
West Virginia	333,400	76.7 (72.6–80.3)	170,400 (154,700–185,700)	51.1 (46.4–55.7)
Wisconsin	1,125,000	79.5 (74.8–83.4)	717,800 (661,500–770,600)	63.8 (58.8–68.5)
Wyoming	111,500	78.5 (74.0–82.4)	63,300 (57,300–69,100)	56.8 (51.4–62.0)
Puerto Rico	734,000	75.6 (72.7–78.2)	332,500 (309,700–356,000)	45.3 (42.2–48.5)
**Overall**	**61,337,100**	**76.2 (75.4–77.0)**	**37,231,600 (36,618,200–37,783,700)**	**60.7 (59.7–61.6)**

**Abbreviation:** CI = confidence interval.

* Women were considered to be at risk for unintended pregnancy unless they reported 1) not being sexually active with a male partner, 2) being currently pregnant or seeking pregnancy, 3) not minding being pregnant, or 4) having had a hysterectomy.

^†^ Women with ongoing or potential need for contraceptive services were defined as women considered to be at risk for unintended pregnancy not using permanent contraception (female sterilization or male partner vasectomy). The number of women with ongoing or potential need for contraceptive services can be used to estimate how many women might seek services.

^§^ Data shown are from 2019, except 2017 data are shown for seven jurisdictions: Alaska, California, District of Columbia, Maine, Nevada, New Jersey, and Texas.

^¶^ Weighted numbers are rounded to the nearest 100.

**TABLE 2:** Estimated numbers and percentages of women aged 18–49 years who were at risk for unintended pregnancy* and had ongoing or potential need for contraceptive services,^†^ by selected sociodemographic characteristics — Behavioral Risk Factor Surveillance System, 45 jurisdictions, 2017–2019^§^

Sociodemographic characteristic	Women aged 18–49 yrs			
	Total no.^¶^	% at risk for unintended pregnancy (95% CI)	No. and % who had ongoing or potential need for contraceptive services	
			No. (95% CI)^¶^	% (95% CI)
**Age group, yrs**				
18–24	13,992,200	71.5 (69.5–73.5)**	9,696,600 (9,402,800–9,990,400)	69.3 (67.2–71.4)**
25–34	20,042,800	74.5 (73.0–75.9)	12,867,500 (12,546,800–13,188,200)	64.2 (62.6–65.8)
35–44	18,901,000	79.5 (78.1–80.8)	10,263,200 (9,960,800–10,546,800)	54.3 (52.7–55.8)
45–49	8,401,100	80.4 (78.6–82.1)	4,435,800 (4,251,000–4,620,600	52.8 (50.6–55.0)
**Race/Ethnicity**				
White, non-Hispanic	30,888,700	78.0 (77.0–78.9)**	18,131,700 (17,791,900–18,471,400)	58.7 (57.6–59.8)**
Black, non-Hispanic	8,764,100	75.3 (73.1–77.5)	5,723,000 (5,503,900–5,933,300)	65.3 (62.8–67.7)
Hispanic	14,526,100	75.5 (73.6–77.2)	8,948,100 (8,643,000–9,238,600)	61.6 (59.5–63.6)
Other	6,305,400	70.8 (67.1–74.2)	3,903,000 (3,657,100–4,136,300)	61.9 (58.0–65.6)
**Insurance coverage**				
Yes	51,112,100	76.2 (75.3–77.1)	30,871,700 (30,360,600–31,382,800)	60.4 (59.4–61.4)
No	9,915,300	76.8 (74.7–78.8)	6,167,300 (5,929,300–6,395,400)	62.2 (59.8–64.5)
**Routine checkup within past yr**				
Yes	43,835,000	76.7 (75.7–77.6)	26,651,700 (26,213,300–27,133,900)	60.8 (59.8–61.9)
No	16,704,300	75.3 (73.6–76.9)	10,056,000 (9,755,300–10,356,700)	60.2 (58.4–62.0)
**Urban-rural status^††^**				
Urban	57,369,500	76.5 (75.6–77.3)**	35,282,200 (34,708,500–35,798,600)	61.5 (60.5–62.4)**
Rural	3,227,300	72.6 (69.6–75.4)	1,639,500 (1,552,300–1,729,800)	50.8 (48.1–53.6)

**Abbreviation:** CI = confidence interval.

* Women were considered to be at risk for unintended pregnancy unless they reported 1) not being sexually active with a male partner, 2) being currently pregnant or seeking pregnancy, 3) not minding being pregnant, or 4) having had had a hysterectomy.

^†^ Women with ongoing or potential need for contraceptive services were defined as women considered to be at risk for unintended pregnancy not using permanent contraception (female sterilization or male partner vasectomy). The number of women with ongoing or potential need for contraceptive services can be used to estimate how many women might seek services.

^§^ 2017: Alaska, California, District of Columbia, Maine, Nevada, New Jersey, and Texas. 2019: Alabama, Arizona, Arkansas, Connecticut, Delaware, Florida, Georgia, Hawaii, Idaho, Illinois, Indiana, Iowa, Kansas, Louisiana, Maryland, Massachusetts, Minnesota, Mississippi, Missouri, Montana, Nebraska, New Mexico, New York, North Carolina, Ohio, Oklahoma, Oregon, Pennsylvania, Rhode Island, South Carolina, South Dakota, Tennessee, Utah, Virginia, West Virginia, Wisconsin, Wyoming, and Puerto Rico.

^¶^ Weighted numbers are rounded to the nearest 100.

** p<0.05 for chi-square test comparing the distribution of the outcome by the sociodemographic characteristic.

^††^ Determined using the 2013 National Center for Health Statistics urban-rural classification scheme for counties. https://www.cdc.gov/nchs/data_access/urban_rural.htm

Among women with ongoing or potential need for contraceptive services, 15.2% used a long-acting reversible method, 25.0% used a short-acting reversible method, 29.5% used a barrier or other reversible method, and 30.3% used no method (Table 3). By jurisdiction, among women with ongoing or potential need for contraceptive services, use of a long-acting reversible method ranged from 6.9% (Puerto Rico) to 36.1% (Utah), use of a short-acting reversible method ranged from 13.7% (Puerto Rico) to 32.9% (Tennessee), use of a barrier or other reversible method ranged from 19.3% (Maine) to 42.6% (District of Columbia), and use of no method ranged from 20.1% (Massachusetts) to 45.8% (Puerto Rico).

**TABLE 3:** Percentages of women aged 18–49 years who had ongoing or potential need for contraceptive services* using specific contraceptive methods, by category of method^†^ or no method and jurisdiction — Behavioral Risk Factor Surveillance System, 45 jurisdictions, 2017–2019^§^

Jurisdiction	% of women aged 18–49 yrs (95% CI)			
	Long-acting reversible method	Short-acting reversible method	Barrier or other reversible method	No method
Alabama	9.6 (7.1–12.9)	25.1 (20.7–30.1)	37.0 (31.9–42.3)	28.3 (23.7–33.3)
Alaska	28.5 (19.6–39.5)	18.6 (11.9–27.8)	31.6 (21.9–43.1)	21.3 (13.9–31.4)
Arizona	13.0 (9.5–17.5)	24.5 (19.3–30.6)	28.3 (22.8–34.4)	34.3 (28.5–40.5)
Arkansas	9.8 (6.2–15.2)	25.2 (19.4–32.0)	30.1 (23.6–37.5)	35.0 (28.6–41.9)
California	15.1 (12.5–18.2)	26.9 (23.1–31.0)	30.4 (26.6–34.4)	27.6 (23.9–31.6)
Connecticut	16.1 (12.5–20.4)	23.8 (19.9–28.2)	31.1 (26.7–35.9)	29.0 (24.8–33.6)
Delaware	12.5 (8.8–17.6)	28.1 (21.8–35.4)	30.1 (23.6–37.6)	29.2 (22.9–36.5)
District of Columbia	11.5 (8.1–15.9)	16.0 (12.3–20.7)	42.6 (37.1–48.3)	29.9 (25.2–35.0)
Florida	12.9 (9.8–16.8)	25.8 (21.2–31.0)	28.9 (23.9–34.4)	32.5 (27.4–38.0)
Georgia	11.2 (8.2–15.0)	25.3 (20.3–31.1)	26.2 (21.3–31.9)	37.3 (31.6–43.3)
Hawaii	16.9 (13.6–20.7)	21.4 (18.0–25.3)	22.3 (18.9–26.1)	39.4 (34.8–44.2)
Idaho	29.4 (23.5–36.1)	22.4 (17.0–28.9)	24.0 (18.4–30.6)	24.2 (19.1–30.2)
Illinois	10.4 (8.1–13.2)	23.8 (20.0–28.1)	35.4 (30.9–40.2)	30.4 (26.0–35.2)
Indiana	14.0 (11.2–17.4)	24.7 (20.8–29.2)	29.7 (25.6–34.2)	31.5 (27.4–35.9)
Iowa	22.3 (19.3–25.7)	28.9 (25.5–32.7)	25.2 (22.0–28.8)	23.5 (20.5–26.8)
Kansas	19.2 (16.3–22.4)	27.7 (24.3–31.4)	23.7 (20.3–27.6)	29.4 (26.0–33.1)
Louisiana	11.1 (8.1–14.9)	25.2 (20.9–30.0)	34.3 (29.4–39.6)	29.4 (25.2–34.0)
Maine	33.6 (26.8–41.2)	26.7 (20.6–33.8)	19.3 (14.3–25.5)	20.4 (15.7–26.2)
Maryland	16.1 (13.5–19.1)	24.4 (21.1–28.1)	33.4 (29.8–37.2)	26.1 (22.9–29.6)
Massachusetts	19.5 (16.3–23.2)	28.6 (24.5–33.0)	31.8 (27.6–36.2)	20.1 (16.6–24.1)
Minnesota	21.9 (19.6–24.4)	28.5 (25.8–31.3)	23.5 (21.0–26.1)	26.2 (23.6–28.9)
Mississippi	10.3 (7.4–14.1)	24.7 (20.1–29.9)	34.1 (29.0–39.7)	30.9 (26.2–36.1)
Missouri	16.5 (12.8–21.1)	27.7 (22.8–33.1)	31.8 (26.6–37.4)	24.1 (19.5–29.3)
Montana	29.2 (24.8–34.1)	23.2 (19.3–27.6)	24.0 (19.9–28.6)	23.6 (19.8–27.9)
Nebraska	17.1 (13.5–21.5)	27.0 (22.7–31.7)	28.7 (24.5–33.4)	27.1 (23.2–31.5)
Nevada	21.2 (15.5–28.3)	22.7 (16.9–29.8)	23.6 (17.3–31.3)	32.5 (25.6–40.1)
New Jersey	12.6 (9.8–16.0)	22.7 (18.6–27.4)	31.1 (26.7–35.8)	33.6 (29.0–38.6)
New Mexico	22.5 (18.0–27.7)	18.0 (13.8–23.1)	27.7 (22.7–33.4)	31.8 (26.5- 37.6)
New York	14.1 (10.2–19.2)	18.1 (14.1–22.9)	33.7 (28.0–39.9)	34.1 (28.4–40.2)
North Carolina	20.0 (15.6–25.1)	24.4 (19.8–29.6)	23.7 (19.2–28.9)	32.0 (26.8–37.6)
Ohio	15.9 (11.7–21.3)	30.8 (25.4–36.9)	23.5 (18.8–29.0)	29.8 (24.5–35.6)
Oklahoma	16.5 (11.9–22.6)	29.7 (23.1–37.1)	26.7 (20.6–33.8)	27.1 (21.4–33.7)
Oregon	30.7 (26.5–35.3)	24.2 (20.5–28.4)	24.8 (21.1–29.0)	20.2 (16.5–24.5)
Pennsylvania	13.9 (11.0–17.3)	30.6 (25.9–35.8)	29.4 (24.5–34.9)	26.1 (21.6–31.1)
Rhode Island	16.6 (12.3–22.1)	31.0 (25.3–37.4)	26.6 (21.0–33.0)	25.8 (20.7–31.7)
South Carolina	11.9 (9.1–15.4)	28.4 (24.0–33.3)	30.8 (26.2–35.8)	28.9 (24.2–34.1)
South Dakota	17.6 (12.7–23.9)	27.7 (21.2–35.2)	26.7 (20.0–34.7)	27.9 (21.4–35.7)
Tennessee	10.5 (7.4–14.7)	32.9 (27.3- 38.9)	28.2 (22.6–34.6)	28.4 (23.2–34.2)
Texas	12.0 (8.6–6.5)	18.3 (14.3–23.3)	30.3 (24.7–36.7)	39.3 (33.2–45.8)
Utah	36.1 (33.0–39.3)	21.9 (19.2–24.8)	21.2 (18.7–24.0)	20.8 (18.3–23.6)
Virginia	17.4 (14.3–21.0)	27.3 (23.1–31.9)	25.1 (21.5–29.0)	30.3 (26.0–34.9)
West Virginia	16.6 (12.2–22.3)	25.9 (20.4–32.2)	27.1 (20.9–34.3)	30.4 (24.7–36.8)
Wisconsin	18.4 (13.8–24.0)	32.4 (26.8–38.4)	25.7 (20.5–31.6)	23.6 (18.8–29.1)
Wyoming	18.7 (13.6–25.1)	23.3 (17.5–30.3)	27.9 (21.6–35.3)	30.1 (23.4–37.8)
Puerto Rico	6.9 (4.7–10.0)	13.7 (10.8–17.4)	33.5 (29.1–38.2)	45.8 (41.1–50.7)
**Overall**	**15.2 (14.4–16.0)**	**25.0 (24.0–26.0)**	**29.5 (28.4–30.6)**	**30.3 (29.2–31.5)**

**Abbreviation:** CI = confidence interval.

* Women with ongoing or potential need for contraceptive services were defined as women considered to be at risk for unintended pregnancy not using permanent contraception (female sterilization or male partner vasectomy). The number of women with ongoing or potential need for contraceptive services can be used to estimate how many women might seek services.

^†^ Categories of contraceptive methods reflect different levels of effort for method initiation and continuation. Long-acting reversible contraception methods include intrauterine devices and contraceptive implants; these methods require the most clinical effort for initiation but require minimal follow-up until time for removal or reinsertion and minimal action by the woman. Short-acting reversible contraception methods include injectables, pills, transdermal patches, and vaginal rings; these methods require less clinical effort for initiation than long-acting reversible methods but require ongoing clinical services and supplies and action by the woman to maintain use. Barrier or other reversible contraception methods included diaphragms, condoms (male or female), withdrawal, cervical caps, sponges, spermicides, fertility-awareness–based methods, and emergency contraception; these methods have little or no need for clinical services for initiation, but require action by the woman or her partner to maintain use.

^§^ Data shown are from 2019, except 2017 data are shown for seven jurisdictions: Alaska, California, District of Columbia, Maine, Nevada, New Jersey, and Texas.

## Discussion

During 2017–2019, six out of 10 (60.7%, approximately 37.2 million women) women aged 18–49 years in the 45 jurisdictions had ongoing or potential need for contraceptive services, with variation observed by jurisdiction, age group, race/ethnicity, and urban-rural status. An estimated 6.2 million women without insurance had ongoing or potential need for contraceptive services and might require publicly funded care. Among women with ongoing or potential need for contraceptive services, nearly one in three (30.3%) was not using contraception at last sexual encounter. Improving contraception access and uptake among these women might have a large effect on meeting reproductive health care needs and reducing unintended pregnancies (*5*). In addition, nearly one in three women (29.5%) used a barrier or other reversible method (i.e., diaphragm, condom, withdrawal, cervical cap, sponge, spermicide, fertility-awareness–based method, or emergency contraception); given lower effectiveness of these methods for preventing unintended pregnancy during typical use compared with long-acting and short-acting reversible methods (*6*), jurisdictions might consider efforts to ensure women’s access to the full range of available contraceptive methods. These efforts might include removing logistic and administrative barriers for contraceptive services and supplies, training providers, increasing provider reimbursement, and increasing consumer awareness of available contraceptive services and method options (*7*).

Jurisdiction-level information on unintended pregnancy risk and need for contraceptive services is important because unintended pregnancy rates vary by jurisdiction (*8*). Variation might be caused by differences in population characteristics (*9*) or differences in the implementation of public health programs and policies to increase contraceptive use. Examples of programs and policies that vary by jurisdiction include participation in a learning community to increase contraception access through a broad range of strategies^§§§^ and leveraging Medicaid coverage to expand eligibility for family planning services.^¶¶¶^

Jurisdiction-level information is also important for planning, enhancing, and evaluating efforts to improve contraception access and services. Contraceptive care clinical performance measures use administrative data to assess health system quality of care (*10*), but these data lack information on unintended pregnancy risk. Data in this report can be used by jurisdictions to calculate the measures**** to better reflect their populations in need. The data provided in this report also have implications for ensuring access to contraceptive services during public health emergencies that have a disproportionate impact on reproductive-aged women or on the delivery of routine clinical services. BRFSS data were previously used to estimate the number of women needing contraceptive services during the 2016 Zika virus outbreak because of concerns about Zika virus–related adverse pregnancy and birth outcomes (*4*). In emergencies that disrupt routine care, jurisdictions can use data on the number of women needing contraceptive services and current contraceptive use to help plan for potential alternative models for service provision (e.g., telehealth/telemedicine) and for ensuring continued access to method supply (e.g., providing or prescribing a 1-year supply of methods that need resupply).^ ††††^

The findings provided in this report are subject to at least four limitations. First, information on contraceptive use was self-reported and might be subject to recall error or social desirability bias. Second, for 21% of women aged 18–49 years, data on the family planning module were missing, mostly a result of incomplete BRFSS interviews. Although the proportion of women for whom these data were missing was generally similar by jurisdiction, a higher proportion of younger women and women who self-identified as non-Hispanic Black or other had missing data. Third, findings apply only to the jurisdictions that implemented the optional family planning module and therefore might not be generalizable to the entire U.S. population of women aged 18-49 years. Finally, nonresponse bias remains a possibility, although the BRFSS weighting methodology adjusts for nonresponse.

Ensuring access to contraceptive services is important to promote reproductive autonomy, prevent unintended pregnancies, and promote optimal and equitable reproductive health. Understanding the need for contraceptive services, including the number of uninsured women who might need publicly supported care, can help jurisdictions plan health care delivery to support women and their partners in choosing whether and when to become pregnant. The data in this report can be used by jurisdictions to estimate the number of women who might seek contraceptive services and to plan and evaluate contraception access policies and programs.

**Summary**

**What is already known about this topic?**

Ensuring access to contraception is important for promoting reproductive autonomy, preventing unintended pregnancies, and promoting optimal and equitable reproductive health.

**What is added by this report?**

Analysis of 2017–2019 Behavioral Risk Factor Surveillance System data from 45 jurisdictions found that 60.7% of women aged 18–49 years had ongoing or potential need for contraceptive services; estimates varied by jurisdiction, age group, race/ethnicity, and urban-rural status. Nearly one third (30.3%) of women with ongoing or potential need were not using any method of contraception at last sexual encounter.

**What are the implications for public health practice?**

Jurisdictions can use the data provided in this report to plan delivery of contraceptive services and evaluate efforts to increase access to contraception.

## Footnotes

* Women were considered to be at risk for unintended pregnancy unless they reported 1) not being sexually active with a male partner, 2) being currently pregnant or seeking pregnancy, 3) not minding being pregnant, or 4) having had a hysterectomy.

† Women with ongoing or potential need for contraceptive services were defined as women considered to be at risk for unintended pregnancy who were not using permanent contraception (female sterilization or male partner vasectomy). The number of women with ongoing or potential need for contraceptive services can be used to estimate how many women might seek services.

§ 2017: Alaska, California, District of Columbia, Maine, Nevada, New Jersey, and Texas. 2019: Alabama, Arizona, Arkansas, Connecticut, Delaware, Florida, Georgia, Hawaii, Idaho, Illinois, Indiana, Iowa, Kansas, Louisiana, Maryland, Massachusetts, Minnesota, Mississippi, Missouri, Montana, Nebraska, New Mexico, New York, North Carolina, Ohio, Oklahoma, Oregon, Pennsylvania, Rhode Island, South Carolina, South Dakota, Tennessee, Utah, Virginia, West Virginia, Wisconsin, Wyoming, and Puerto Rico.

¶
https://www.cdc.gov/brfss/data_documentation/index.htm

** 2017 questions are listed in Module 17: Preconception Health/Family Health (https://www.cdc.gov/brfss/questionnaires/pdf-ques/2017_BRFSS_Pub_Ques_508_tagged.pdf). 2019 questions are listed in Module 23: Family Planning (https://www.cdc.gov/brfss/questionnaires/pdf-ques/2019-BRFSS-Questionnaire-508.pdf).

†† Twenty-one percent of women aged 18–49 years had missing data on being at risk for unintended pregnancy status, primarily caused by incomplete BRFSS interviews administered via mobile telephone rather than missing data specifically on the family planning module questions.

§§ Women at risk for unintended pregnancy who did not specify the type of contraception that they used or reported “other method” (4.8%) were excluded from estimates of women with ongoing or potential need for contraceptive services and from estimates of contraceptive use by method category. Write-in responses were not available for women who responded “other method”; previous evaluation of BRFSS contraceptive use data indicates that these methods are a mix of permanent and reversible methods. https://www.cdc.gov/mmwr/volumes/65/wr/mm6530e2.htm

¶¶ Age was ascertained by the answer to the question, “What is your age?” Race/ethnicity was ascertained by the answer to the question, “Which one or more of the following would you say is your race?” Health insurance coverage was ascertained by the answer to the question, “Do you have any kind of health care coverage, including health insurance, prepaid plans such as HMOs, or government plans such as Medicare, or Indian Health Service?” Routine checkup within the past year was ascertained by the answer to the question, “About how long has it been since you last visited a doctor for a routine checkup?” Urban-rural status was determined using the 2013 National Center for Health Statistics urban-rural classification scheme for counties.

*** Contraceptive method use at last sexual encounter was ascertained by the answers to the questions, “The last time you had sex with a man, did you or your partner do anything to keep you from getting pregnant?” (2019) or “What did you or your partner do the last time you had sex to keep you from getting pregnant?” (2017).

††† Response rates for the included jurisdictions in 2019 ranged from 37.3% (New York) to 73.1% (South Dakota) (https://www.cdc.gov/brfss/annual_data/2019/pdf/2019-response-rates-table-508.pdf). Response rates for the included jurisdictions in 2017 ranged from 31.4% (California) to 49.3% (Maine). https://www.cdc.gov/brfss/annual_data/2017/pdf/2017-response-rates-table-508.pdf

§§§ The learning community was convened by the Association of State and Territorial Health Officials in collaboration with CDC, Centers for Medicare & Medicaid Services, and the U.S. Department of Health and Human Services Office of Population Affairs. Examples of strategies to improve contraceptive use included improving provider awareness and training; addressing logistical, stocking, and administrative barriers; improving reimbursement and financial sustainability; and ensuring adequate service locations. https://www.astho.org/Programs/Maternal-and-Child-Health/Increasing-Access-to-Contraception/

¶¶¶
https://www.guttmacher.org/state-policy/explore/medicaid-family-planning-eligibility-expansions

****
https://opa.hhs.gov/research-evaluation/title-x-services-research/contraceptive-care-measures/most-or-moderately

††††
https://rhntc.org/resources/what-family-planning-providers-can-do-meet-client-needs-during-covid-19?utm_source=eNews&utm_campaign=March
